# Preserved Perspective Taking in Free Indirect Discourse in Autism Spectrum Disorder

**DOI:** 10.3389/fpsyg.2021.675633

**Published:** 2021-07-07

**Authors:** Juliane T. Zimmermann, Sara Meuser, Stefan Hinterwimmer, Kai Vogeley

**Affiliations:** ^1^Department of Psychiatry, Faculty of Medicine and University Hospital Cologne, University of Cologne, Cologne, Germany; ^2^Institute of Language and Literature I, University of Cologne, Cologne, Germany; ^3^Institute of Language and Literature – Linguistics, University of Wuppertal, Wuppertal, Germany; ^4^Institute of Neuroscience and Medicine, Cognitive Neuroscience (INM-3), Research Centre Juelich, Juelich, Germany

**Keywords:** autism spectrum disorder (ASD), perspective taking, free indirect discourse (FID), perspectival centers, mentalizing, theory of mind (ToM)

## Abstract

Perspective taking has been proposed to be impaired in persons with autism spectrum disorder (ASD), especially when implicit processing is required. In narrative texts, language perception and interpretation is fundamentally guided by taking the perspective of a narrator. We studied perspective taking in the linguistic domain of so-called Free Indirect Discourse (FID), during which certain text segments have to be interpreted as the thoughts or utterances of a protagonist without explicitly being marked as thought or speech representations of that protagonist (as in direct or indirect discourse). Crucially, the correct interpretation of text segments as *FID* depends on the ability to detect which of the protagonists “stands out” against the others and is therefore identifiable as implicit thinker or speaker. This so-called “prominence” status of a protagonist is based on linguistic properties (e.g., *grammatical function*, *referential expression*), in other words, the perspective is “hidden” and has to be inferred from the text material. In order to test whether this implicit perspective taking ability that is required for the interpretation of *FID* is preserved in persons with ASD, we presented short texts with three sentences to adults with and without ASD. In the last sentence, the perspective was switched either to the more or the less prominent of two protagonists. Participants were asked to rate the texts regarding their naturalness. Both diagnostic groups rated sentences with *FID* anchored to the less prominent protagonist as less natural than sentences with *FID* anchored to the more prominent protagonist. Our results that the high-level perspective taking ability in written language that is required for the interpretation of *FID* is well preserved in persons with ASD supports the conclusion that language skills are highly elaborated in ASD so that even the challenging attribution of utterances to protagonists is possible if they are only implicitly given. We discuss the implications in the context of claims of impaired perspective taking in ASD as well as with regard to the underlying processing of *FID*.

## Introduction

One of two key symptoms of autism spectrum disorder (ASD) refers to social communication and interaction disturbances (American Psychiatric Association, 2013). One explanation for these phenomena is an impaired ability to take the perspective of others (Baron-Cohen et al., 1985; Frith et al., 1991), also referred to as *Theory of Mind* (*ToM*; Premack and Woodruff, 1978) or *mentalizing* (Fonagy et al., 2004; Frith and Frith, 2006). This impairment has often been demonstrated in language-based tasks with children with ASD^1^ (Baron-Cohen et al., 1985; Baron-Cohen, 1989; Leslie and Thaiss, 1992; Swettenham, 1996; Hutchins et al., 2012; Begeer et al., 2014). Adolescents or adults with ASD and normal intelligence usually pass comparable false-belief tasks designed to probe second-order *ToM* tasks as successfully as control participants (Bowler, 1992; Happé, 1994; Ponnet et al., 2004; Scheeren et al., 2013). In these tasks, participants are prompted with explicit questions regarding the mental state of a protagonist. These tasks probe an explicit and, hence, better accessible type of perspective taking. On the other hand, tasks that require a more implicit type of perspective taking appear to be problematic for adolescents or adults with ASD, even under conditions of normal intelligence. This is especially revealed when participants are asked to not only infer a protagonist’s mental state, but also to provide reasons for their attributions (Ozonoff et al., 1991; Bowler, 1992; Happé, 1994; Dziobek et al., 2006; Callenmark et al., 2014), similarly, when eye movement is measured to assess overt attention in false-belief tasks (Senju et al., 2009; Schneider et al., 2013; Schuwerk et al., 2015). Impairments are also visible when inferring a protagonist’s mental state based on photo or video material (Baron-Cohen et al., 2001; Ponnet et al., 2004; Dziobek et al., 2006), which might explain why participants with ASD rely in their impressions formation of others significantly more on verbal than on non-verbal information (Kuzmanovic et al., 2011).

The interpretation of an utterance does not only depend on the linguistic content and its context, but also and essentially on the person of the speaker. An utterance of a sentence containing a so-called predicate of personal taste (e.g., “licorice is tasty”; Lasersohn (2005)) might be true for one, but not for another person. Furthermore, utterances including deictic expressions referring to persons (“I”, “you”), places (“here,” “there”) and/or time (“now”, “then”) can only be successfully interpreted in their context (i.e., speaker, reader/listener, location, time). In contrast to spoken language, written text does not always allow for an unambiguous identification of the speaker or perspectival center. It has been proposed (Zeman, 2017) that processing of so-called *Free Indirect Discourse* (*FID*; Banfield (1982)) shares an important aspect with perspective taking involved in *ToM* as operationalized in many false belief tasks, namely the ability to identify and differentiate between separate viewpoints at the same time. Importantly, we believe that *FID* processing differs from false-belief tasks insofar as perspective taking in FID is implicit. While in false-belief tasks commonly mastered by adults with ASD and normal intelligence the instruction to take a perspective is explicit, in *FID* it is implicit as readers are not instructed to take the perspective of a certain protagonist, but rather switch perspectives automatically in order to reach a sensible interpretation. Harris and Potts (2009) showed that certain context-sensitive markers have the potential to alter text interpretation so that perspective is shifted away from the first-person narrator to a competing protagonist. Kaiser (2015) demonstrated that *FID* cues increase perspectival-center-oriented text interpretation. However, these studies do not consider contexts in which multiple protagonists can serve as potential anchors for the utterance in *FID* mode.

In *FID*, utterances or thoughts are to be ascribed to a protagonist without explicitly mentioning her/him as the source of the utterance or thought. In the following example: “When Thomas entered the pub a guy in a black coat punched him right in the face with his bare hand. Ouch, how that hurt!” the reader will most likely understand that the last sentence expresses the experience of Thomas, whereas it is much less likely that the punching guy complains about his hand hurting. Without any explicit linguistic markers (e.g., quotation marks), *FID* is commonly indicated by the use of more subtle signals (Banfield, 1982; Steube, 1985), such as an exclamative (“Ouch!”) or a judgmental statement (“that hurt”). Often, *FID* can only be interpreted correctly when certain parts of the sentence such as deictic adverbials of space and time or expressions such as “Ouch” are anchored to the protagonist’s perspective (e.g., it is Thomas who feels pain, not the narrator) while others such as pronouns and tenses are anchored to the narrator’s perspective (e.g., for Thomas, being punched does not hurt in the past, but in the present) (Schlenker, 2004; Eckardt, 2014). In other words, the interpretation of *FID* requires the identification of the implicit anchor for a specific thought or utterance and, hence, taking the perspective of one protagonist as opposed to another (Example *1*).

(A) *On Monday morning Jaqueline was running to the classroom in a hurry. In the hallway she bumped into her classmate_m_. Now she would have to go to the nurse with that clumsy oaf.*
(B) *On Monday morning Arne was running to the classroom in a hurry. In the hallway he bumped into his classmate_f_. Now she would have to go to the nurse with that clumsy oaf.*
(C) *On Monday morning Arne was running to the classroom in a hurry. In the hallway he bumped into his classmate_f_. She went to the nurse with him.*
(D) *On Monday morning Arne was running to the classroom in a hurry. On the hallway he bumped into his classmate_f_. He went to the nurse with her.*

Example 1: *One variation of a scenario as it appeared in our study in the four different conditions A, B, C, and D. The last sentence of item A and B is an instance of FID that needs to be anchored to one of the two protagonists of the preceding sentences to be interpreted sensibly. Items C and D do not contain FID. All texts were presented in German, followed the same structure and were similar in style. German words may denote a specific gender (e.g., classmate, German: “Klassenkameradin,” or “Klassenkamerad”), indicated with “f” (female), and “m” (male).*

In our study we follow a so-called prominence-based account for *FID* anchoring (Hinterwimmer, 2019), according to which the prominence status is the key for perspective ascription. Prominence refers to the property of a linguistic element (e.g., a syllable, a word, a sentence) as “standing out” in contrast to a group of similar elements (Streefkerk, 2002; Himmelmann and Primus, 2015). The protagonist who is more prominent in terms of *grammatical function* and *type of referential expression* (i.e., the expression we use to refer to an object or a person, e.g., “Thomas”, “he”) is more plausible as the anchor for *FID* than a competing protagonist (Hinterwimmer and Meuser, 2019). Based on the assumption that *FID* anchoring requires implicit perspective taking, these findings indicate that *FID* anchored to the more prominent protagonist is perceived as more natural and therefore receives higher ratings on a scale indicating acceptability by test persons, because it is easier or more common to take the prominent protagonist’s perspective. For the purpose of our study we systematically varied *grammatical function* and *type of referential expression* as influential factors for a protagonist’s prominence status. In the hierarchy of *grammatical functions*, a subject is more prominent than an indirect object, which is in turn more prominent than a direct object and so forth (Himmelmann and Primus, 2015). With respect to *referential expression* a protagonist that is familiar to the reader is more prominent than a protagonist that is unfamiliar (Jasinskaja et al., 2015). We make use of these prominence-lending features by claiming that a protagonist who is introduced with her/his first name and picked up by a pronoun in subject position is easier identified as the perspectival center for *FID* ascription than a competing protagonist who is introduced with an indefinite noun phrase in object position, which was already shown to be the case in an acceptability rating study by Hinterwimmer and Meuser (2019).

So far, it has not been clarified which particular linguistic types of perspective taking are consistently affected in exactly what way in ASD during speech and language production and perception, especially with regard to the shifting of perspectival centers. While *FID* perception has not been investigated in ASD so far, the production and perception of *referential expressions* has been studied already. While people with ASD and normal intelligence perform well in verbal perspective taking tasks, subtle differences indicate problems with respect to *ToM* in language production. The general population tends to adjust their choice of *referential expressions* to the listener or reader (i.e., depending on the context, we choose to substitute names with pronouns; Achim et al. (2017)). Adults with ASD use more full noun phrases during narratives when they could use pronouns instead, while, on the other hand, they use more pronouns when full noun phrases would be less ambiguous and hence would make it easier to understand the narration (Colle et al., 2008). This finding could indicate a reduced *ToM* in ASD with regard to the listener (Colle et al., 2008). This behavior has, however, not consistently been reported (Arnold et al., 2009). In a perception study investigating spatial perspective taking, participants with ASD showed unimpaired performance and neural activation comparable to a control group during the perception of written text referring to two people by their first names in third person, namely the participant and another person. On the other hand, when the task required perspective shifts induced by references to the participant as “you”, performance decreased and neural patterns differed compared to the control group (Mizuno et al., 2011).

In our web-based study, we investigate for the first time the perception of shifting perspectival centers by means of *FID* in written language in adults with ASD. This implicit form of perspective taking might not be as easily accomplished by adults with ASD as by adults without ASD. Therefore, we expected to identify difficulties in *FID* processing in persons with ASD. In our study, participants judged the naturalness of sentences including *FID* anchored to protagonists of different prominence status. Based on the idea that texts in which the required perspective taking is easier to accomplish are linked to higher naturalness ratings, and considering the reported perspective taking difficulties in people with ASD in implicit *ToM* tasks, we anticipated lower naturalness ratings in people with ASD for texts associated with implicit perspective taking, especially if the required perspective shift is an unusual one. More specifically, we pursued the following hypotheses:

H1: The difference between naturalness ratings for texts including *FID* (here: condition A) and ratings for texts not including *FID* (here: condition D) will be greater in the ASD group in comparison to the control group.
H2: The difference between naturalness ratings for texts including *FID* anchored to the less prominent protagonist (here: condition B) and ratings for texts including *FID* anchored to the more prominent protagonist (here: condition A) will be greater in the ASD group in comparison to the control group. If H1 is supported, differences between ratings for condition A and B might play a minor role.

## Materials and Methods

### Participants

Only participants who were monolingual native speakers of German were included in the study. For the ASD group, we recruited 45 adults with ASD via a mailing list of the Outpatient Clinic for Autism in adulthood at the University Hospital of Cologne. Of these, 41 participants had a diagnosis of Asperger syndrome (F.84.5 according to ICD-10), four participants indicated a diagnosis of high-functioning autism, one of these a diagnosis of childhood autism (F.84.0). For the control group, we recruited 45 adults without a diagnosis of ASD via the intranet of the University Hospital Cologne, publicly accessible notice boards and personal contacts (Table 1 for sample characteristics).

**TABLE 1:** Sample characteristics.

	Gender	Age	WST	BDI-II	AQ	EQ
ASD (*N* = 45)	25 men 20 women	20 - 82 years men: *M* = 48.2 (*SD* = 13.9) women: *M* = 42.6 (*SD* = 10.9)	*M* = 112.3 (*SD* = 10.00)	M = 13.8 (*SD* = 9.30)	*M* = 42.5 (*SD* = 4.25)	*M* = 13.8 (*SD* = 5.95)
Control (*N* = 45)	25 men 20 women	20 - 80 years men: *M* = 47.7 (*SD* = 14.7) women: *M* = 41.0 (*SD* = 12.3)	*M* = 111.0 (*SD* = 9.35)	M = 8.2 (*SD* = 6.27)	*M* = 15.5 (*SD* = 6.60)	*M* = 47.0 (*SD* = 12.5)

In the group of participants with ASD, 25 of 45 participants with ASD reported that they had experienced depressive episodes. Participants with ASD indicated the following medication for the treatment of psychological, psychiatric and neurological conditions: antidepressants (15 participants), mood stabilizer (1), neuroleptic medication (1). Control participants indicated no history of neurological or psychiatric disorders. No psychotropic medication was reported by any participant in the control group. Scores for verbal intelligence as measured with the *Wortschatztest* (*WST*, Schmidt and Metzler (1992)) indicated average or above-average verbal intelligence in all participants (Table 1) and did not differ between groups (two-samples *t*-test, *t*(88) = −0.63, *p* = 0.530). Depressive symptoms measured with the *Beck depression inventory II* (*BDI-II*, Beck et al. (1996)) were significantly higher in participants with ASD than in control participants (Table 1, Welch two-samples *t*-test, *t*(77.1) = −3.34, *p* = 0.001), with symptoms ranging from none to clinically relevant symptoms in both groups. Scores indicating autistic traits measured with the *autism quotient* (*AQ*, Baron-Cohen et al. (2001)) were significantly higher in participants with ASD compared to the control group (Table 1, Welch two-samples *t*-test, *t*(75.2) = −23.08, *p* < 0.001). Scores indicating empathetic traits measured with the *empathy quotient* (*EQ*, Baron-Cohen and Wheelwright (2004)) were significantly lower in participants with ASD compared to the control group (Table 1, Welch two-samples *t*-test, *t*(63.1) = 16.15, *p* < 0.001).

### Text Material

We presented short German narrative texts with three sentences each. We developed 24 different scenarios with a common theme. Each scenario was varied systematically in four different conditions, resulting in a total of 96 different texts. The conditions varied with respect to utterances with *FID* (conditions A and B; see example *1*) or without *FID* in neutral story continuation (conditions C and D; see example *1.* See Table 2 for an overview of experimental conditions and the Supplementary Material for the complete list of texts). The content of the utterance with *FID* was thematically ambiguous with respect to two protagonists that were both potential candidates for the perspectival center, i.e., the thought presented as *FID* in the last sentence of the text could plausibly be linked to either one of the two protagonists, if the pronoun in the third sentence did not allow for unambiguous resolution. The utterance with *FID* thus varied with respect to the pronoun that indicated which one of the two protagonists was the anchor of the thought.

**TABLE 2:** Overview of experimental conditions; “P” stands for “protagonist”.

Condition	Subject in S1	Subject in S2	Subject/Perspective in S3
A: *FID*, prominent	P1	P1	P1
B: *FID*, non-prominent	P2	P2	P1
C: Control, subject change	P2	P2	P1
D: Control, no subject change	P2	P2	P2

In the first sentence (S1) of each text, one of two protagonists was introduced by a proper name in subject position, and an explicit reference to the past (e.g., “Monday morning”) was included. In the second sentence (S2) the protagonist introduced in S1 was picked up with a personal pronoun in subject position interacting with a second protagonist who was referred to with a full noun phrase and who was anchored to the first protagonist with a possessive pronoun (e.g., “her/his classmate”). Contrary to the English equivalent, the German noun phrases used in our stimuli were each linked to a specific gender (female/male). Therefore, both protagonists (P1 and P2) differed with regard to gender so that the *FID* in S3 could only reasonably be anchored to either P1 or P2.

The target sentence (S3) in condition A and condition B was an utterance in *FID* mode. It featured three indicators of *FID*: (i) a temporal adverbial referring to the present (e.g., “now,” “today”) or an immediate or close future (e.g., “soon,” “tomorrow”) contrasting with the temporal adverbials in S1, (ii) a verb in subjunctive II mode (e.g., “would”), and (iii) a colloquial term or qualitative noun (e.g. “clumsy oaf”). Conditions C and D served as control conditions. Unlike the target sentence S3 in *FID* conditions, S3 in control conditions did not feature any markers of *FID*. The target sentence continued the story in neutral narrative story mode. Control condition D continued with P1 in subject position while in condition C, P2 was the subject. Thus, the two neutral conditions resembled the test conditions regarding content and syntactic structure.

In order to investigate the anchoring of *FID* we manipulated our texts with regard to the two protagonists in three different ways, with respect to (i) the grammatical function of the first expression referring to them (subject or object), (ii) the number of references (two or three), and iii) the type of referring expression (first name and pronouns or noun phrase and colloquial term). Based on previous findings (Hinterwimmer and Meuser, 2019) we predicted that in control participants *FID* anchored to the more prominent protagonist, i.e., the one in subject position, referred to with their first name and picked up by an adequate personal pronoun (condition A), would more likely be accepted as the perspectival center of a sentence in *FID* than the competing protagonist who was introduced with a noun phrase in object position in the second sentence (condition B). Texts in condition A should thus be rated more natural than texts in condition B.

As our manipulation of the utterance in *FID* mode involved a change or continuation of the subject with respect to one of the two protagonists, we included two control conditions C and D to account for the effect of subject change based on differences in referential chains: In condition C, the pronoun in subject position of the final sentence picked up the object of the preceding sentence, while in condition D, it picked up the subject. If texts of condition C would be rated comparable to texts of condition D, we might conclude that differences between the two *FID* conditions cannot be explained by (dis)continuity of referential chains alone. As both story continuations were equally coherent in terms of content, both control conditions C and D should be equally acceptable.

We included 40 filler texts similar to the 96 target texts in length and complexity (see Supplementary Material). In order to mask our manipulation, some filler texts were deliberately designed to yield low acceptability by an odd choice of pronouns, i.e., in the last sentence, a personal pronoun was used which referred back to an inanimate entity that occurred in object position in the previous sentence in which a personal pronoun was used to refer to the protagonist (“[…] He ate the cake_m_. He was made of marzipan.”). All four conditions were equally distributed across four lists so that every participant was presented with only one condition (A, B, C, or D) of each of the 24 scenarios and the total set of 40 filler texts, resulting in 64 texts in total that were presented to each participant in random order.

### Procedure

The experiment was programmed and presented on Ibex farm, a platform for online experiments (Drummond, 2020). It was conducted in accordance with the Declaration of Helsinki (World Medical Association, 2013) and approved by the Ethics Committee of the Medical Faculty of the University of Cologne. Informed consent was obtained from all participants prior to participation. Demographic data and information on clinical diagnoses and medication was collected. In the following rating, participants were instructed to judge the naturalness of the third sentence in the context of the first two sentences of each presented text on a scale from 1 (labeled “very unnatural”) to 7 (labeled “very natural”). For each text, presentation duration including response time was limited to 25 seconds. After completing the naturalness ratings, participants were given the opportunity to report what they noticed about the task in an open format. Psychological questionnaires were obtained afterward: *WST, AQ, EQ, BDI-II*. The *BDI-II* was included due to the high incidence of depressive symptoms in persons with ASD (Ghaziuddin et al., 2002). Finally, participants had the opportunity to make assumptions with regard to the aims of the study. The whole procedure engaged participants for approximately one hour. They were debriefed and compensated for their participation with a gift voucher of ten Euro.

### Analysis

Data was analyzed using R (R Core Team, 2019) in RStudio (RStudio Team, 2016). We fitted Bayesian ordinal models using the *brms* package (Bayesian Regression Models using Stan, v2.10.0; Bürkner (2017); Bürkner and Vuorre (2019)). Factors were sum-coded. Weakly informative priors were used for group-level effects as well as for random intercepts (normal distribution; mean = 0; standard deviation = 2) and fixed intercepts (normal distribution; mean = 4, i.e., the center of the rating scale; standard deviation = 2). Estimated parameters are reported in terms of posterior means and 95% credibility intervals. To investigate the evidence for or against the investigated effects, we compared models by calculating Bayes factors applying the *bayesfactor_models* function from the *bayestestR* package (Makowski et al., 2019) which uses bridge sampling (Gronau et al., 2020). All models ran with four sampling chains of 12,000 iterations each including a warm-up period of 2,000 iterations.

#### Models

To test hypothesis 1 and thus the influence of *FID* and diagnosis, i.e., to identify differences between the groups regarding naturalness ratings for texts with *FID* and ratings for comparable texts without *FID*, a Bayesian ordinal mixed model was fitted to the ratings from conditions A and D. Fixed effects used in the model were *FID*, *group* and their interaction. Additionally, we included random intercepts and slopes for the factor *subject* as well as random intercepts for *text*. To test hypothesis 2 and thus the influence of protagonist prominence and diagnosis, i.e., to identify differences between the groups regarding naturalness ratings for texts with *FID* anchored to the more prominent protagonist and ratings for texts with *FID* anchored to the less prominent protagonist, a Bayesian ordinal mixed model was fitted to the data of the acceptability ratings for conditions A and B. Fixed effects used in the model were *prominence*, *group* and their interaction. Additionally, we included random intercepts and slopes for the factor *subject* as well as random intercepts for *text*. To demonstrate that a subject shift toward the less prominent protagonist does not in general lead to lower ratings, but only in *FID* conditions, we ran a Bayesian ordinal mixed model for naturalness ratings of our control conditions that did not include *FID*, i.e., neutral condition C including a subject shift toward the less prominent protagonist and neutral condition D not including a subject shift. Fixed effects used in the model were *subject shift*, *group* and their interaction. Additionally, we included random intercepts and slopes for the factor *subject* as well as random intercepts for *text*. Because texts in conditions C and D are minimally different, which is not the case for texts in conditions A and B, differences between C and D are not fully equivalent to differences in A and B. Thus, the resulting conditions do not allow to test our hypotheses in a single model. Therefore, we addressed our hypotheses in separate models. To investigate evidence for or against the presence of effects, we additionally ran the following models for comparison with each of these models: the respective null model not including the group level factors; the model including only one of either factor; and the model including the linear combination of both factors. We report respective Bayes factors of model comparisons and follow the interpretation by Jeffreys (1939).

#### Explorative Analyses

We carried out correlational explorative analyses to identify possible relationships between the naturalness ratings and parameters we collected in addition to the ratings, i.e., psycho(patho)logical measures and age. To account for individual rating behavior, we standardized the ratings for each participant applying a rank-based non-linear transformation to the ratings of all four conditions, which for each participant results in normally distributed rating values centered around zero. Influences due to individually different scale use are therefore minimized. We investigated correlations across and within the two groups for the difference between ratings for condition A and D with our parameters (i.e., *AQ, EQ, BDI-II, WST*, AQ-scores for the subscales *attention switching*, *communication* and *imagination, age*). Differences between conditions were calculated by subtracting the standardized ratings for condition D from the standardized ratings for condition A. Likewise, correlations were investigated between our parameters and the difference between the standardized ratings for conditions A and B. We report Pearson correlation coefficients or Spearman’s rank correlation coefficients reaching significance at the 5% confidence-level.

## Results

**FIGURE 1:**
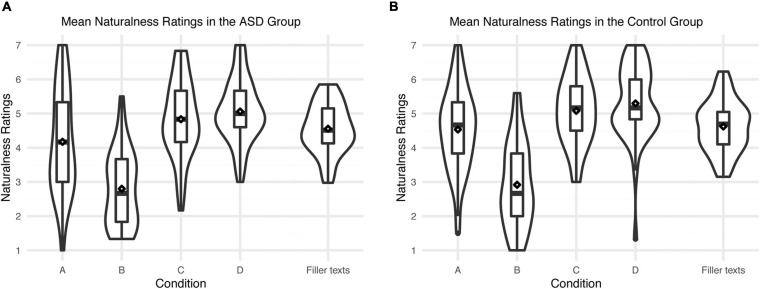
Mean naturalness ratings for the four conditions and filler texts in the ASD group **(A)** and the control group **(B)**. Diamonds indicate means.

In general, texts in conditions A and B including *FID* were rated less natural (condition A: *M* = 4.34, *SD* = 2.23; condition B: *M* = 2.85, *SD* = 1.91) than conditions C and D not including *FID* (condition C: *M* = 4.96, *SD* = 2.00; condition D: *M* = 5.17, *SD* = 2.04). Across the whole sample naturalness ratings for texts in condition A were higher than for texts in condition B. Ratings did not show any statistically meaningful difference between both diagnostic groups. See Figure 1 for an overview of mean ratings per condition for both diagnostic groups.

### Comparison of FID Condition A and Neutral Condition D

*FID* affected the ratings by lowering the units on the latent rating scale by 0.48 (95% CI = [−0.64, −0.32]). An ASD diagnosis showed a general tendency to lower the ratings (*b* = −0.18, 95% CI = [−0.43, 0.07]), however, the influence of a diagnosis on the ratings was smaller than that of *FID*. The interaction of *FID* and *group* hardly affected the ratings (*b* = *−*0.03, 95% CI = [*−*0.36, 0.29]). Model comparisons indicated extreme evidence only for an influence of *FID*. They further revealed moderate evidence for the absence of a *group* effect. Strong evidence was found against an interaction effect. Bayes factors for the models in comparison to the null model: full model: BF > 1000; model with linear combination: BF > 1000; model including only the factor *group*: BF = 0.16; model including only the factor *FID*: BF > 1000. We further investigated rating patterns in the two groups using the *marginal_effects* function from the *brms* package. The model results and the rating behavior within the two groups did not support the assumption of *FID* affecting rating behavior of the two groups differently. Thus, hypothesis 1 is not supported by our data.

Correlation analyses (see Table 3) showed that *age* was positively correlated with the difference between standardized ratings for conditions A and D in the control group (*r*_P_ = 0.39, *p* = 0.008; ASD group: *r*_P_ = *−*0.17, *p* = 0.277). This indicates that the difference between ratings for sentences including *FID* and ratings for sentences without *FID* decreases with age in the control group.

**TABLE 3:** Correlations between psycho(pathological) measures and rating differences for compared conditions.

		AQ	EQ	BDI-II	WST	AQ attention switching	AQ communication	AQ imagination	age
A minus D (Difference bt. rank-based-standardized ratings)	Both groups	*r_S_* = −0.07 (*p* = *0.531*)	*r_S_* = 0.02 (*p* = 0.836)	*r_S_* = −0.08 (*p* = 0.480)	*r_P_* = −0.13 (*p* = 0.217)	*r_S_* = −0.08 (*p* = 0.431)	*r_S_* = −0.04 (*p* = 0.710)	*r_S_* = −*0.04* (*p* = 0.695)	*r_S_* = 0.12 (*p* = 0.260)
	ASC	*r_S_* = −0.19 (*p* = *0.218*)	*r_S_* = −0.11 (*p* = *0.489*)	*r_P_* = 0.02 (*p* = 0.889)	*r_P_* = −0.24 (*p* = *0.111*)	*r_S_* = −0.06 (*p* = 0.708)	*r_S_* = −0.15 (*p* = *0.314*)	*r_S_* = −0.16 (*p* = 0.295)	*r_P_* = −0.17 (*p* = 0.277)
	Control	*r_P_* = 0.18 (*p* = 0.224)	*r_P_* = −0.04 (*p* = *0.778*)	*r_S_* = −0.03 (*p* = *0.856*)	*r_P_* = 0.00 (*p* = *0.980*)	*r_S_* = 0.03 (*p* = *0.848*)	*r_S_* = 0.28 (*p* = *0.065*)	*r_S_* = 0.23 (*p* = *0.135*)	*r_P_* = 0.39 (*p* = 0.008)*
A minus B (Difference bt. rank-based-standardized ratings)	Both groups	*r_S_* = −0.10 (*p* = 0.327)	*r_S_* = 0.05 (*p* = *0.666*)	*r_S_* = −0.01 (*p* = *0.943*)	*r_P_* = −0.09 (*p* = *0.400*)	*r_S_* = −0.23 (*p* = *0.031*)*	*r_S_* = −0.11 (*p* = 0.297)	*r*_*S*_ = −*0.06* (*p* = 0.593)	*r_S_* = 0.11 (*p* = *0.302*)
	ASC	*r_S_* = −0.06 (*p* = 0.686)	*r_S_* = −0.15 (*p* = 0.327)	*r_P_* = −0.09 (*p* = *0.577*)	*r_P_* = −0.16 (*p* = *0.308*)	*r_S_* = −0.35 (*p* = 0.018)*	*r_S_* = −0.18 (*p* = 0.235)	*r_S_* = 0.16 (*p* = *0.298*)	*r_P_* = −0.06 (*p* = *0.709*)
	Control	*r_P_* = *0.06* (*p* = 0.695)	*r_P_* = −0.15 (*p* = *0.320*)	*r_S_* = 0.26 (*p* = 0.080)	*r_S_* = 0.01 (*p* = 0.958)	*r_S_* = 0.01 (*p* = 0.929)	*r_S_* = 0.29 (*p* = 0.050)	*r_S_* = 0.09 (*p* = 0.554)	*r_P_* = 0.30 (*p* = 0.045)*

*P-values <0.05 are marked with an asterisk.*

### Comparison of FID Conditions A and B

Reducing protagonist prominence generally affected the ratings by lowering the units on the latent rating scale by 0.85 (95% CI = [*−*1.06, *−*0.65]). ASD diagnosis lowered the ratings. However, this tendency was smaller than the effect of protagonist prominence (*b* = −0.12, 95% CI = [−0.37, 0.14]). The interaction showed that reduced *prominence* tended to result in higher ratings in the ASD group in comparison to the control group (*b* = 0.17, 95% CI = [*−*0.23, 0.57]). Model comparisons indicated extreme evidence for an influence of reduced protagonist prominence. They further revealed moderate evidence for an absence of a *group* effect as well as for an absence of an interaction effect (Bayes factors for the models in comparison to the null model: full model: BF > 1000; model with linear combination: BF > 1000; model including only the factor *group*: BF = 0.1; model including only the factor *prominence*: BF > 1000). The model results and the rating behavior within the two groups did not support the assumption of prominence affecting rating behavior of the two groups differently. Thus, hypothesis 2 is not supported by our data.

Comparable to the correlation analysis for the comparison of conditions A and D, correlation analyses showed that *age* was positively correlated with the difference between standardized ratings for conditions A and B in the control group only (*r*_P_ = 0.30, *p* = 0.045; ASD group: *r*_P_ = −0.06, *p* = 0.709). This indicates that the difference between ratings for *FID* anchored to the less prominent protagonist and ratings for *FID* anchored to the more prominent protagonist decreases with age in the control group. Moreover, correlations of our psycho(patho)logical measures with the difference between standardized ratings for conditions A and B showed a statistically significant correlation across the sample (*r*_S_ = *−*0.23, *p* = 0.031), which appears to mainly be driven by the sample with ASD: In this group, the scores of the *AQ* subscale *attention switching* were moderately negatively correlated with the difference between standardized ratings for conditions A and B (*r*_S_ = *−*0.35, *p* = 0.018). This indicates that participants with ASD reporting more problems regarding attention switching tend to give less divergent ratings for conditions A and B.

### Comparison of Neutral Conditions C and D

The analysis suggests that a subject shift alongside the respective *referential expression* lowered the ratings (*b* = *−*0.16, 95% CI = [*−*0.30, *−*0.02]). The factor *group* showed a tendency to also lower the ratings (*b* = *−*0.15, 95% CI = [*−*0.39, 0.09]). The interaction hardly influenced the ratings (*b* = 0.03, 95% CI = [*−*0.25, 0.32]). Model comparisons, however, showed no reliable evidence for the presence of any of these effects and tendencies in our data, as indicated by Bayes factors favoring the null model over all other models while at the same time lacking robustness (Bayes factors for the models in comparison to the null model: full model: BF < 0.001; model with linear combination: BF = 0.01; model including only the factor *group*: BF = 0.13; model including only the factor *subject shift*: BF = 0.08).

### Further Explorative Analyses

Visual inspection of naturalness ratings distributions suggested bimodality. To test if bimodality was present in our data, we tested for each ratings distribution in each condition in each group the deviance from unimodality. We used the R package *diptest* (v0.75-7; Mächler, 2015) which applies Hartigan’s dip test (Hartigan and Hartigan, 1985). The results indicated that unimodality was not given at a 95%-confidence level in condition C in the ASD group as well as in condition B and C in the control group. In the remaining conditions, unimodality was not given at a 90%-confidence level. Therefore, the visual impression was corroborated by the test. We performed a median split of the data to identify if there was a difference between people that tend to give higher ratings and people that tend to give lower ratings. To this end, we split the groups into two subgroups (high-rating subjects and low-rating subjects) based on their ratings in condition D, which we set as the reference condition for this analysis, because it does not contain *FID* nor a subject shift. We then ran the models already introduced above again with the additional factor *subgroup* (high-rating vs low-rating) along its interaction terms with the other factors.

The results of this analysis of subgroups showed that the negative effect of *FID* on the ratings in condition A as opposed to condition D seemed to be mediated mostly by participants who rated high in condition D. Most importantly, this pattern did not differ statistically in the two subgroups of both the ASD and the control group. Further, the results indicated that *FID* anchoring to the less prominent protagonist lead to lower ratings in all subgroups. Most importantly, this pattern did not differ statistically for the ASD subgroups and the control subgroups, indicating that the tendency for high or low ratings is more fundamental than the differential response behavior due to diagnostic groups.

### Participants’ Feedback

Most participants found the texts – at least to some degree – confusing, stylistically clumsy, illogical, and/or grammatically wrong. Several participants perceived a lack of coherence due to sudden subject or perspective shifts (supposedly in the case of neutral and *FID* texts) or due to the third sentence containing ambiguous reference (supposedly in the case of filler texts). Six participants (five with ASD) noticed and/or found the shifts of perspective in the third sentence confusing (supposedly with regard to *FID* texts), referring to this factor as “perspective shift”, “shifting perspective” to the protagonist that the story was not about, “shift of (emotional) narrative perspective”, “brutal shift of the narrative perspective,” “illogical perspective,” and “ambiguous perspective.” The markers we used to indicate *FID* were partly perceived as unnatural, both by people with ASD and control participants. Not only markers of *FID* were mentioned in the feedback, but also our markers of prominence. Three participants with ASD reported problems with the interpretation of task instructions for the judgment of naturalness or a difficulty to integrate naturalness regarding the narrative style and naturalness regarding the content into a comprehensive rating of naturalness. Some participants felt torn between what to base their rating on, e.g., whether they should base their rating on what would be considered natural with regard to the behavior of the protagonists and the content of the story, or rather on whether this was a narrative form that could naturally be encountered. Three participants in the ASD group reported that they found it hard to make a decision within the time limit.

## Discussion

The aim of this study was to investigate the perception of *FID* and prominence in the context of *FID* in participants with ASD. In contrast to our hypotheses, we did not observe any difference in the performance between persons with ASD in comparison to unaffected control persons. The first focus related to hypothesis H1 was the acceptability of *FID* in the ASD group compared to the control group based on naturalness ratings of sentences including *FID* (condition A) as opposed to neutral sentences not containing *FID* (condition D). The second focus related to hypothesis H2 was the study of the difference between naturalness of *FID* anchored to a more prominent protagonist as opposed to a less prominent one (conditions A and B). Contrary to both hypotheses, the ratings were comparable and did not differ between the diagnostic groups, neither with respect to the presence of *FID* (conditions A vs. D, hypothesis H1) nor with respect to anchoring to more or less prominent protagonists (conditions A vs. B, hypothesis H2). Technically speaking, the factor *group* did not improve the adequacy of the statistical model. Taken together, both hypotheses had to be rejected.

### FID Processing

Across the whole sample, naturalness ratings were lower for sentences with *FID* (conditions A and B) compared to sentences without *FID* (conditions C and D). This result is in accordance with findings of a previous study in the general population in which test items with *FID* received lower ratings in general. Additionally, in that study test items with *FID* anchored to the perspective of a more prominent protagonist yielded higher acceptability ratings than test items with *FID* anchored to the perspective of a less prominent protagonist (Hinterwimmer and Meuser, 2019). We could replicate this effect in our study, further supporting the notion of prominence as a relevant factor for anchoring *FID*. In our control analysis in neutral conditions, i.e., non-*FID* sentences, we showed that a subject shift as manipulated via *grammatical function* and *referential expression* shows a tendency, but not a reliable decrease, to lower acceptability ratings when pronouns need to be resolved. This indicates that the effect of *prominence* reported above cannot be explained by subject shift alone.

Interestingly, we found that the control sample as well as the ASD sample could both be divided into two subgroups with different rating tendencies. Persons who generated high ratings in condition D were more strongly affected by *FID*, whereas the effect of prominence for *FID* anchoring was comparable across subgroups. The explanation for these subgroups’ behavior might be trivial: High-raters might tend to rate the acceptability of texts with *FID* worse compared to low-raters, because they have more rating variance available to indicate their perception. However, individual factors might also play a role such as different perspective taking abilities (Kaiser and Cohen, 2012) or language dexterity. Interestingly, this pattern was visible across the control and the ASD sample, which further underlines that rating patterns for *FID* in general and prominence-dependent *FID* anchoring in particular are not affected in ASD.

With respect to the processes involved, we propose that the anchoring of *FID* depends both on perspective taking as well as on linguistic markers, more specifically, on perspective taking and the ascription of the perspectival center of a text which in turn depends on the linguistic notion of prominence (Hinterwimmer, 2019). That leaves two strategies to anchor an utterance in *FID* mode which may be both involved: (i) the reader may ascribe an utterance in *FID* mode to the perspectival center of the text and/or (ii) they may ascribe an utterance in *FID* mode to a protagonist based on linguistic markers i.e., prominence-lending cues.

### Influence of Age

Another interesting observation was the correlation of the ratings with age. In the control group, we report a relationship of *age* with the naturalness-ratings for sentences with *FID* as opposed to sentences without *FID*, in other words, both types of sentences are rated more similar with increasing *age*. The same relationship was found for *age* and the naturalness-ratings for *FID* anchored to the less prominent protagonist as opposed to *FID* anchored to the more prominent protagonist. This might be related to a cognitive decline that also involves language comprehension (Burke and Shafto, 2007) as well as referential processing such as in anaphor resolution based on problems recalling contextual information (Light and Capps, 1986). *FID* processing might be affected in older participants in a similar fashion, since it requires anchoring to a protagonist previously introduced in the context. Furthermore, tracking of protagonist prominence relations has been suggested to be affected in older adulthood (Hendriks et al., 2014). More generally, studies in older participants show that *ToM* abilities decrease with *age* across different experimental tasks (Henry et al., 2013).

In contrast to these aforementioned aspects that putatively explain the reduced *FID* sensitivity in older participants, greater linguistic experience could on the other hand allow for easier processing (Crocker and Keller, 2006) which could in turn lead to an increased acceptance of sentences in *FID* mode in older people, but also to easier processing of *FID* anchoring to less prominent protagonists as opposed to more prominent ones. Additionally, psycho-affective changes associated with higher *age* might play a role, such as a more positive mindset in general (Carstensen et al., 2010). Finally, *age*-associated cognitive decline affecting text processing may be compensated for by other abilities that improve with *age* such as crystallized abilities like vocabulary, or change with *age* such as allocation of attention during reading (Stine-Morrow et al., 2008).

Notably, we did not observe any such relationship with age in persons with ASD. Research on aging in people with ASD is sparse in general and often inconsistent (Happé and Charlton, 2012; Howlin and Magiati, 2017). While some cognitive abilities seem to decline in ASD similarly to the general population (Howlin and Magiati, 2017), others are less affected than in the general population, such as working memory (Lever et al., 2015) or align with control participants with age resulting in comparable abilities in both groups, such as *ToM* abilities (Lever and Geurts, 2016). Thus, different lifetime trajectories of cognitive abilities responsible for *FID* processing might explain the different rating behavior in ASD with increasing age.

### Conceptual Issues

#### Theory of Mind (ToM)

One key capacity associated with perspective taking is *ToM*, the ability to ascribe mental states to oneself and others, also closely related to language (Gernsbacher and Yergeau, 2019). In adolescents or adults with ASD, language abilities can partly explain performance in *ToM* tasks (Peterson and Miller, 2012; Lombardo et al., 2015) and strange stories tasks (Abell and Hare, 2005). Based on clinical diagnoses and *WST* performances, we can make sure that participants with ASD did not display any substantial language problems.

Our findings are in concordance with research showing that text-based second-order *ToM* abilities in high-functioning adults and adolescents with ASD are largely unimpaired (Bowler, 1992; Happé, 1994; Ponnet et al., 2004; Scheeren et al., 2013; Schuwerk et al., 2015; Murray et al., 2017). However, in contrast to our data, second-order implicit *ToM* abilities have indeed been reported to be affected in ASD in some studies (Ponnet et al., 2004; Dziobek et al., 2006; Murray et al., 2017). Our data show that persons with ASD are not compromised in this specific *FID* task. Language-related *ToM* impairments have been argued to be subtle (Colle et al., 2008). The most obvious interpretation seems to be that adult persons with ASD with good verbal intelligence are obviously able to learn the complex processes of perspective taking that can be expressed via written language, even if implicit perspective taking is required.

However, it is also possible that our purely behavioral measures in this web-based study were not sensitive enough to identify group differences. Previous studies have shown difficulties associated with second-order *ToM* tasks despite correct task responses, e.g., regarding the causal reasoning about others’ mental states (Ozonoff et al., 1991; Bowler, 1992; Happé, 1994; Dziobek et al., 2006), eye movements (Senju et al., 2009; Scheeren et al., 2013; Schneider et al., 2013; Schuwerk et al., 2015; Murray et al., 2017) as well as regarding the attribution of belief which has been shown to not happen automatically (Senju et al., 2009) and to be more difficult for adults with ASD than for control participants (Bradford et al., 2018). Future studies on *FID* in ASD should therefore also include either a non-text-based *ToM* task to assess if persons with ASD show second-order *ToM* impairments in other domains or a *FID* component that requires faster responses, possibly as a task in an ongoing interaction with another person.

#### Embodiment

There is strong evidence that readers tend to create complex mental models of the presented situation including the protagonists’ experiences (e.g., Zwaan and Radvansky (1998)) for which also spatial grounding is a necessary prerequisite (Beveridge and Pickering, 2013). Listeners or readers might even embody the protagonists to re-experience their actions (Kiefer and Pulvermüller, 2012) which possibly facilitates empathizing with them (van Berkum, 2019). Furthermore, participants also adopt a story’s timeline as they need more time to remember events if more time has passed in the story’s timeline (Zwaan, 1996; Carreiras et al., 1997). If taking the perspective of a protagonist is accompanied by *embodiment*, *FID* anchoring could possibly be embodied, too. Spatial perspective taking related to embodiment seems to play a role in FID interpretation as indicated by its correlation with FID sensitivity (Kaiser and Cohen, 2012). Furthermore, embodiment has been shown to be relevant for *referential expressions*: In written texts, processing of singular second person pronouns (Brunyé et al., 2011; Gianelli et al., 2011) as well as third person pronouns, but the latter only with spatial anchoring (Gianelli et al., 2011), are usually accompanied by *embodiment* in control participants. This effect seems to happen automatically (Ditman et al., 2010).

If embodiment is indeed involved in *FID* anchoring, the use of third-person pronouns such as in our texts might pose an obstacle for identifying its influence on *FID* anchoring, because embodiment seems to be limited in this case (Gianelli et al., 2011). In a study investigating different text styles on spatial grounding, Salem et al. (2017) found that *FID* alongside spatial anchors presented within the text did not increase self-reported identification with the protagonist nor did it affect spatial perspective taking of participants.

A disturbance of embodiment was proposed to offer an explanation for problems adults with ASD have with certain mentalizing tasks especially in the spatial domain (Pearson et al., 2013). But embodiment does not appear to be necessary, depending on the task, mental rotation processes could be employed (Pearson et al., 2013; Conson et al., 2015). In such a spatial task, participants with ASD showed mostly unimpaired performance when written texts referred to the participant or the other person with first names (Mizuno et al., 2011). In our study, we assume that participants did not make use of any such strategies related to visual perspective taking, as we have not systematically varied spatial information in our texts.

#### Executive Control

Basic abilities required for perspective taking are inhibitory control (Brown-Schmidt, 2009; Wardlow, 2013) and working memory capacity (Lin et al., 2010; Wardlow, 2013). Both of these executive abilities have been reported to be impaired in persons with ASD (Demetriou et al., 2018; Habib et al., 2019).

The ability to shift between or integrate different perspectives requires the balanced inhibition of one or more of potentially competing perspectives (MacWhinney, 2000; Frith and de Vignemont, 2005). Competing tasks demanding *executive control* hinder the correct selection of perspectives (Qureshi et al., 2010). Schwarzkopf et al. (2014) hypothesized for the visual domain that persons with ASD do in fact implicitly take the perspective of others. However, to decode behaviorally relevant interpretations of the perspective of another person, an attentional shift away from their own perspective toward another person’s perspective is necessary, which might be less easily accomplished in ASD (Schwarzkopf et al., 2014). Our explorative correlation analysis suggests that people with ASD reporting more problems with attention switching tend to give less divergent ratings for conditions A and B. One related explanation could be that impaired attention switching might lead to less perspective taking and to reduced sensitivity for cognitively effortful *FID* anchoring as opposed to effortless *FID* anchoring. However, because we did not investigate executive functions, these claims are speculative. Potentially, implicit methods could in principle reveal processing differences in ASD while behavior is otherwise unimpaired (e.g., Bradford et al. (2018)).

*Executive control* is not only relevant for the shifting of perspective, but also for keeping track of a story or a conversation, and thus for establishing and maintaining prominence relations, accordingly, working memory abilities have been shown to have a positive effect on the cognitive maintenance of shared conversational information or “common ground” in ASD (Schuh et al., 2016). Other abilities impaired in ASD such as planning and fluency (Demetriou et al., 2018) might play a role in predicting, updating and maintaining common ground, and thus the tracking of prominence relations. Our results suggest largely preserved abilities regarding inhibiting less prominent anchors for the interpretation of *FID*, of storing information in working memory to predict upcoming information and of shifting attention toward the different perspectival centers to interpret *FID*. Thus, in our task, participants with ASD appear to track prominence relations comparable to control participants.

## Limitations

One limitation of the study was that we did not test any of the capacities discussed under the umbrella terms of *ToM, embodiment* or *executive control*. Our results therefore offer a first insight into how *FID* is processed at the behavioral level, but cannot yet inform us about potential differences regarding their underlying cognitive processes.

Our web-based study did not allow us to measure reaction times. Considering the issue of response time, further studies investigating persons with ASD should potentially allow for longer time frames for the participants’ response or use different methods like self-paced reading to accommodate different needs regarding the duration of stimulus presentation. To stimulate embodied text processing and thus increase perspective taking, longer and more vivid texts might be helpful (MacWhinney, 2000).

## Conclusion

In this paper, we have shown that implicit perspective taking based on verbal abilities in the context of *FID* is fully preserved in ASD. We replicated the results of previous studies in healthy control persons (Hinterwimmer and Meuser, 2019) that the prominence status of protagonists in written short stories affects acceptability judgments of *FID* anchored to these protagonists. Our results suggest intact processing of *FID* in adults with ASD. We speculate that a possible impairment with respect to second-order *ToM* in ASD can possibly be compensated or can be successfully dealt with in the verbal domain when conventionalized linguistic operations are applied. Further investigations of *FID* interpretation in ASD will benefit from additional measures beyond naturalness ratings, such as implicit measures like reaction time, eye movement, neurophysiological measures or neuroimaging that might shed light on specific processes involved in perspective taking such as *ToM*, *embodiment* or *executive control*, possibly with a focus on discerning attention switching abilities and conventionalized linguistic operations. With respect to treatment, this result implies that interventions can potentially make use of these language-based resources when focusing on impairments, such as inferring mental states from photos or video animations (Baron-Cohen et al., 2001; Ponnet et al., 2004; Dziobek et al., 2006) or beliefs (Bradford et al., 2018) and intentions (Ozonoff et al., 1991; Bowler, 1992; Happé, 1994; Dziobek et al., 2006).

## Data Availability Statement

The datasets presented in this article are not readily available because data handling is restricted to the collaborating institutes by our ethics proposal to secure sensitive data such as psycho(patho-)logical data. Requests to access the datasets should be directed to JZ, juliane.zimmermann@uk-koeln.de.

## Ethics Statement

The studies involving human participants were reviewed and approved by Ethics Commission of Cologne University’s Faculty of Medicine. The patients/participants provided their written informed consent to participate in this study.

## Author Contributions

SH and KV contritbuted to theoretical discussions. JZ and SM designed and conducted the study. JZ analyzed the data and wrote the first manuscript version with contributions from SM. All authors read and modified the manuscript several times. All authors listed have made a substantial, direct and intellectual contribution to the work, and approved it for publication.

## Conflict of Interest

The authors declare that the research was conducted in the absence of any commercial or financial relationships that could be construed as a potential conflict of interest.

## Footnotes

1 The use of “person-first” terminology in the context of ASD is controversial (Kenny et al., 2016; Vivanti, 2020). We apply a clinical perspective that focuses on common symptoms (or the absence thereof), which has been argued to be adequate depending on the context (Tepest, 2021).
